# Global Transcriptional Dynamics of Diapause Induction in Non-Blood-Fed and Blood-Fed *Aedes albopictus*

**DOI:** 10.1371/journal.pntd.0003724

**Published:** 2015-04-21

**Authors:** Xin Huang, Monica F. Poelchau, Peter A. Armbruster

**Affiliations:** Department of Biology, Georgetown University, Washington, D.C., United States of America; Colorado State University, UNITED STATES

## Abstract

**Background:**

*Aedes albopictus* is a vector of increasing public health concern due to its rapid global range expansion and ability to transmit Dengue virus, Chikungunya virus and a wide range of additional arboviruses. Traditional vector control strategies have been largely ineffective against *Ae*. *albopictus* and novel approaches are urgently needed. Photoperiodic diapause is a crucial ecological adaptation in a wide range of temperate insects. Therefore, targeting the molecular regulation of photoperiodic diapause or diapause-associated physiological processes could provide the basis of novel approaches to vector control.

**Methodology/Principal Findings:**

We investigated the global transcriptional profiles of diapause induction in *Ae*. *albopictus* by performing paired-end RNA-Seq of biologically replicated libraries. We sequenced RNA from whole bodies of adult females reared under diapause-inducing and non-diapause-inducing photoperiods either with or without a blood meal. We constructed a comprehensive transcriptome assembly that incorporated previous assemblies and represents over 14,000 annotated dipteran gene models. Mapping of sequence reads to the transcriptome identified differential expression of 2,251 genes in response to diapause-inducing short-day photoperiods. In non-blood-fed females, potential regulatory elements of diapause induction were transcriptionally up-regulated, including two of the canonical circadian clock genes, *timeless* and *cryptochrome 1*. In blood-fed females, genes in metabolic pathways related to energy production and offspring provisioning were differentially expressed under diapause-inducing conditions, including the oxidative phosphorylation pathway and lipid metabolism genes.

**Conclusions/Significance:**

This study is the first to utilize powerful RNA-Seq technologies to elucidate the transcriptional basis of diapause induction in any insect. We identified candidate genes and pathways regulating diapause induction, including a conserved set of genes that are differentially expressed as part of the diapause program in a diverse group of insects. These genes provide candidates whose diapause-associated function can be further interrogated using functional genomics approaches in *Ae*. *albopictus* and other insects.

## Author Summary

The mosquito, *Aedes albopictus*, is an aggressive human biter capable of transmitting Dengue virus, Chikungunya virus and at least 22 additional viruses that cause human illness. Over the last 30 years, this mosquito has spread from its native Asian range to all continents except Antarctica. Efforts to control this mosquito have met with limited success. Photoperiodic diapause refers to the ability of insects to measure day length (photoperiod) as a cue for initiating developmental arrest (dormancy) in order to survive unfavorable seasonal conditions such as winter. Photoperiodic diapause is a crucial ecological adaption that enables *Ae*. *albopictus* and other medically important mosquitoes to inhabit temperate environments and spread across broad geographic ranges. Here, we identify genes that exhibit changes in expression levels (up-regulation or down-regulation) in association with the induction of photoperiodic diapause in *Ae*. *albopictus*. Some of these genes, based on their known biological function in other organisms, are implicated in regulating photoperiodic diapause and represent exciting targets for novel vector control strategies based on genetic or chemical disruption of this important adaptation.

## Introduction

Dengue virus (DENV) and Chikungunya virus (CHIKV) are (re)emerging arboviruses transmitted primarily by the mosquitoes *Aedes aegypti* and *Aedes albopictus*. Estimates of annual DENV infections range from 50–390 million [1,2]. Although levels of CHIKV infection are much lower, a 2005–2006 CHIKV outbreak on La Réunion island in the Indian Ocean infected over 266,000 individuals [3], and was followed by an outbreak in northern Italy in 2007 [4]. This latter event generated considerable concern because it represents the first temperate outbreak of CHIKV, which had previously been restricted to tropical areas. CHIKV has recently spread to the Caribbean islands and South America [5,6], and local transmission of both DENV and CHIKV has recently occurred in peninsular Florida [7,8] and Europe [9]. Although *Ae*. *aegypti* has historically been considered the primary vector of both DENV and CHIKV, *Ae*. *albopictus* has been implicated as the primary vector in at least five DENV outbreaks between 2001–2010, including one in the temperate location of Croatia [9,10], and *Ae*. *albopictus* was also the primary vector of the CHIKV outbreak on La Réunion, which coincided with a CHIKV mutation from Alanine to Valine at position 226 of the E1 viral envelope protein which confers increased transmission efficiency of CHIKV by *Ae*. *albopictus* [11,12].

*Ae*. *albopictus* is an aggressive human biting mosquito that has spread from its native Southeast Asian range to all continents except Antarctica over the last 30 years [13]. This mosquito is a competent vector of 22 other arboviruses in addition to DENV and CHIKV [14]. Because vaccines and drug treatments are not available for DENV, CHIKV and most other arboviruses, vector control has been the most effective strategy for controlling these diseases. However, traditional vector control approaches such as insecticides and source reduction have been largely ineffective against *Ae*. *albopictus* [15], in part because this ecological generalist occupies such a wide range of container types as larval habitats [15,16]. Thus, novel approaches to suppressing this vector are urgently needed.

Photoperiodic diapause is a pre-programmed developmental arrest in response to the token environmental stimulus of photoperiod. Photoperiodic diapause is a crucial adaptation to survival during the unfavorable conditions of winter in a wide variety of temperate insects, including many important vectors of human disease [17–19]. Because diapause is essential for overwinter survival in temperate habitats, identifying novel targets for genetic or chemical disruption of diapause or diapause-associated physiological processes could provide new tools to augment traditional vector control approaches. Genetic approaches to vector control are becoming increasingly feasible [20–22], and the diapause response represents an attractive target for genetic control strategies because diapause-disrupting genetic constructs could be effectively spread by released males during early-spring and mid-summer when there is no requirement for diapause, but then would have a lethal effect when winter arrives. Also, the diapause response involves the modulation of a wide variety of fundamental physiological processes. Therefore, determining the molecular regulation of these processes can provide additional targets for novel vector control strategies.

Kostal [23] defines five eco-physiological phases of the diapause program. First, the diapause induction phase is characterized by perception of the environmental token stimuli well in advance of the adverse seasonal conditions. Diapause induction is followed by a preparation stage when direct development continues but certain physiological processes occur to help the organism prepare for the actual diapause (arrest) stage. Next, during diapause initiation, direct development ceases and metabolic rates are reduced. During the actual diapause phase, the state of diapause is maintained, even under conditions favorable for growth and reproduction. After a certain period of time or in response to chilling or other unknown factors [23], diapause is terminated and direct development can be resumed. Recent studies utilizing RNA sequencing (RNA-Seq) in a range of species have begun to elucidate global transcriptional profiles of diapause preparation [24,25], initiation, maintenance [26,27] and termination [27,28]. These studies have significantly increased understanding of the molecular basis of this crucial ecological adaptation. Results of these studies emphasize that diapause is a dynamic physiological and metabolic process [29]. For example, in the preparation phase organisms need to accumulate extra nutrients to survive the long months of diapause through the winter [29]. Lipid metabolism stands out as a common molecular theme across species and at different stages of diapause, indicating that energy conservation and utilization before, during and after diapause are essential for the organisms’ survival [29].

Despite increasing knowledge of the molecular regulation of diapause preparation, maintenance and termination, the molecular mechanisms regulating the more “upstream” stage of diapause induction remain much less well understood. The molecular mechanisms of diapause induction have been well studied in *Bombyx mori* [30–32], and to a lesser extent in the mosquito *Culex pipiens* [33–35]. These studies confirm the fundamental importance of hormonal regulation during diapause induction [36,37]. However, the mechanism by which organisms measure and interpret photoperiod remains completely unresolved and controversial. Some researchers have argued that the circadian clock provides the mechanistic basis of photoperiodic time measurement [38–40], while others have argued that components of the circadian clock, specifically *timeless*, might function as a component of an “hourglass” interval timer that can measure photoperiod independent of its role in the circadian clock [41,42].

*Ae*. *albopictus* is an outstanding model to study the molecular underpinnings of photoperiodic diapause. *Ae*. *albopictus* undergoes a well characterized photoperiodic diapause, which can be easily and consistently induced in the laboratory by short-day photoperiods [43–45]. In the diapause response of *Ae*. *albopictus*, the photosensitive pupal or adult female perceives the signal of short day length and subsequently produces offspring in which the pharate larva enters diapause inside the chorion of the egg. In *Ae*. *albopictus*, diapause eggs are both more resistant to cold temperatures [46] and desiccation [47,48] than non-diapause eggs. Diapause eggs are also larger and contain ca. 30% more total lipids than non-diapause eggs [49]. Genomic resources for the study of diapause include previously established extensive gene expression profiles of *Ae*. *albopictus* across multiple life stages in the diapause program [24–26,50]. In addition, the published genome sequence of *Ae*. *aegypti* [51], a closely related species, provides a valuable genomic resource.

In this study, we utilized an RNA-Seq approach to examine global transcriptional profiles of diapause induction in the adult *Ae*. *albopictus* females that had either received a blood meal or had not received a blood meal. Previous experiments with *Ae*. *albopictus* have demonstrated that the “photoperiodic switch” triggering the transition from producing non-diapause eggs to diapause eggs can occur before a blood meal [52]. Thus we hypothesized that genes in molecular pathways related to photoperiod interpretation and the early stages of hormonal regulation would exhibit differential expression under diapause-inducing vs. non-diapause-inducing photoperiods in females without a blood meal. We also hypothesized that genes in molecular pathways related to nutritional provisioning of diapause eggs would exhibit differential expression in blood-fed females under diapause conditions. We first constructed a comprehensive transcriptome for *Ae*. *albopictus* across multiple life stages by combining sequences from the current study of the adult stage with those from previous studies of pre-adult stages. We then analyzed differential gene expression by performing read mapping back to the composite transcriptome. Our analysis of differential gene expression proceeded in the following three steps: A) we validated key transcriptional responses to a blood meal under both diapause-inducing and non-diapause-inducing conditions, B) we identified potential regulatory elements during diapause induction by analyzing differential gene expression in females reared under diapause-inducing vs. non-diapause-inducing photoperiods without a blood meal, C) we identified genes and pathways related to maternal provisioning of diapause eggs by analyzing differential gene expression in females reared under diapause-inducing vs. non-diapause-inducing photoperiods with a blood meal. Our results emphasize that diapause is an adaptive metabolic plasticity that involves dramatic changes in transcriptional activity and reinforces our previous hypothesis that a conserved set of genes has contributed to the evolution of diapause in divergent insect lineages.

## Methods

### Insect rearing, tissue preparation and RNA extraction

A laboratory colony of *Ae*. *albopictus* was established from over 200 individuals collected as larvae from more than 10 used tires located at a recycling center in Manassas, Virginia. The colony was maintained for two generations under a non-diapause-inducing long-day (LD) photoperiod of 16L:8D at 21°C and approximately 80% relative humidity as described previously [53,54].

**Fig 1:**
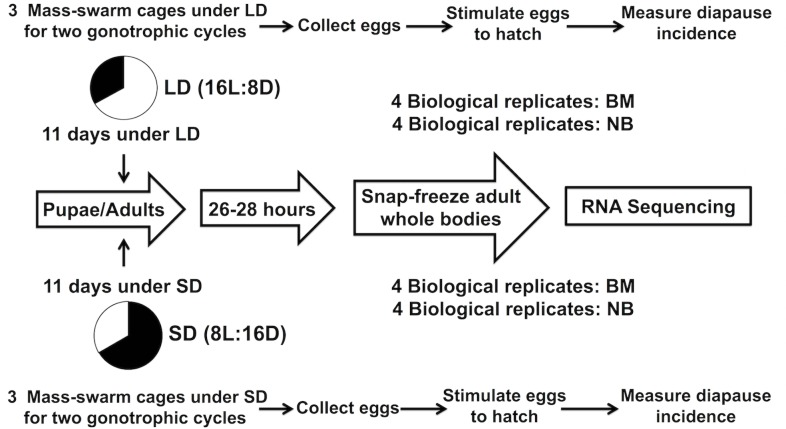
Experimental design for RNA-Seq experiment and diapause incidence measurements. SD indicates diapause-inducing short-day conditions, and LD indicates non-diapause-inducing long-day conditions (see text). BM indicates females that received a blood meal, and NB indicates females that did not receive a blood meal.

An overview of the experimental design is presented in Fig 1. Details of the general workflow for producing tissue and RNA samples under diapause and non-diapause conditions can be found in Poelchau et al. [44]. Briefly, the F_3_ laboratory generation larvae were reared under LD conditions described above. Upon pupation, females were transferred into replicate adult cages (= biological replicates) of approximately 60 females per cage. Eight adult cages were established under a diapause-inducing short-day (SD) photoperiod (8L:16D) and eight adult cages were established under a non-diapause-inducing LD photoperiod (16L:8D) (16 total cages). Additionally, three adult mass-swarm cages containing 30 male and 30 female mosquitoes were established under both SD and LD photoperiods to measure diapause incidence (six total mass-swarm cages).

This experiment was designed to examine the effects of diapause-inducing short-day photoperiods on gene expression of females both with and without a blood meal (Fig 1). Therefore, adult female cages were maintained under SD and LD conditions for 11 days which is sufficient to produce an unambiguous diapause (SD) vs. non-diapause (LD) signal [52]. After 11 days, within each photoperiod treatment (SD and LD), four replicate cages received a blood meal and four replicate cages did not receive a blood meal. Females were blood fed on a human host to repletion between Zeitgeber times (ZT) 3–4h. The Georgetown University Institutional Review Board (IRB) determined that mosquito blood feeding did not qualify as human subjects research and thus did not require IRB approval. However, the blood feeding protocol was approved by the Georgetown University Occupational Health and Safety Committee. Shortly after blood feeding, females were CO_2_ anesthetized and only females with a swollen abdomen and a visible blood meal were retained for the blood meal treatment. The non-blood-fed cages were maintained in parallel to the blood-fed cages. Twenty-six to twenty-eight hours after blood feeding, at ZT 6–8h, female whole bodies from both blood-feeding treatments under both LD and SD photoperiods were snap-frozen in liquid nitrogen and stored at -80°C for RNA extraction. Twenty-six to twenty-eight hours after a blood meal at 21°C is expected to correspond to slightly before the peak of the transcriptional response to a blood meal [55], when the proteolytic activity in the midgut is still increasing [56]. Adult whole bodies were chosen for RNA extraction instead of specific tissues in order to obtain a global transcriptional profile of diapause induction because very little is known about the molecular physiology of diapause induction in adult females of species that undergo embryonic diapause (but see [30,31]), and literally nothing has been published on this topic in mosquitoes [19]. Prior to RNA extraction, the blood bolus was dissected out from the female body in RNAlater (Sigma Aldrich, St. Louis, Missouri), and the female bodies were stored in RNAlater at 4°C for approx. 24 hours. Next, the female whole bodies were ground in TRI Reagent (Sigma Aldrich, St. Louis, Missouri) and RNA was extracted according to manufacturer’s instructions. Residual DNA in the RNA samples was removed by Turbo-DNA free (Ambion, Austin, Texas). Integrity assessment of total RNA was performed by fluorometry on an RNA chip (Bioanalyzer 2100, Agilent Technologies, Santa Clara, California). Four biological replicates from each of four experimental treatments (i.e., SD blood fed and non-blood fed, LD blood fed and non-blood fed) were submitted for sequencing, resulting in 16 sequenced RNA libraries (see *Sequencing* below).

Diapause incidence measurements were performed following methods described in [57]. Briefly, females from the three mass-swarm cages maintained under SD and the three mass-swarm cages under LD were allowed to blood feed to repletion on a human host two to three days after eclosion. Females were blood fed a second time, 6 days later, to collect eggs over multiple gonotrophic cycles. Four days after the first blood meal, a small brown jar half-filled with approximately 50mL of deionized water and lined with unbleached paper towels was placed into each cage to stimulate oviposition. Six days after the first blood meal, paper towels with eggs were removed, maintained under SD conditions for 48–72h, and then gently air-dried. Egg collection was performed every two to three days for two weeks. Egg papers were stored at approximately 80% relative humidity under SD for at least seven days before they were exposed to a hatching stimulus. Eggs are not photosensitive and the uniform treatment under SD at the egg stage does not induce diapause in *Ae*. *albopictus* [43]. Eggs ranging from one to two weeks of age were stimulated to hatch by submersion in water. The number of hatched larvae was recorded and the egg papers were re-dried. This procedure was repeated 7 days later and then the eggs were bleached [58] to record the number of embryonated but unhatched (= diapause) eggs. Diapause incidence (DI) was calculated as DI = (number of embryonated unhatched eggs)/(number of hatched eggs + number of embryonated unhatched eggs) [52,57].

### Sequencing

Illumina paired-end mRNA-Seq library construction was performed by the Institute for Genome Sciences at the University of Maryland according to the TruSeq RNA sample preparation kit (Version 2) (Illumina Inc., San Diego, California). The 16 libraries were individually bar-coded [59] according to manufacturer’s instructions and equally split for paired-end sequencing on two flow-cell lanes of an Illumina HiSeq 2000 sequencer (average insert size = 203 bp; read length = 101 bp). Raw reads are available in NCBI’s short read archive under BioProject accession PRJNA268379.

### Read cleaning

The procedures for assembly and annotation of the transcriptome described below, as well as the procedures for read mapping to quantify differential gene expression, have been described in detail in previous publications from this laboratory [25,50]. Raw reads were first screened using ssaha2 [60] and the UniVec database (accessed July 7th, 2013) to remove vector sequences, adapters, linkers, and primers commonly used in cloning cDNA or genomic DNA as well as *Ae*. *albopictus* rRNA sequences (GenBank accession L22060.1). The cutoff for sequence removal was 95% identity and an alignment score of 18. In addition, Illumina sequencing multiplexing adapters were identified using ssaha2, with 100% percent identity and an alignment score of 18 as the cutoff for removal. In all cases, contaminated reads were removed along with their read mates in a pair. After ssaha2 screening, cleaned reads were further filtered using SolexaQA V2.2 to retain contiguous reads longer than 50 bp with phred quality scores higher than 30.

### Digital normalization and *de novo* assembly

We performed one round of digital normalization [61] to reduce the number of redundant reads and the computational requirements for assembly. This method has previously been shown to drastically reduce the memory necessary for assembly while maintaining the assembly quality [50]. Digital normalization was performed with the default parameters, including a kmer coverage cutoff of 20 and a kmer size of 20. *De novo* assembly was performed using Trinity [62] (released February 25th, 2013) with default parameters. A minimum kmer coverage of 2 was used to reduce memory requirements with hundreds of millions of read pairs.

### Reference-based re-assembly and annotation

In order to produce a comprehensive transcriptome assembly for *Ae*. *albopictus*, the contigs from the current experiment (adult) were combined with previous contigs from *de novo* assemblies of pre-adult stages, including mature oocytes [24], developing embryos [25] and pharate larvae [26,50] using a modified Scaffolding Translation Mapping approach [63] (see Table 1). To eliminate contig redundancy within each life stage, before assembling a comprehensive transcriptome, *de novo* assembled contigs were clustered within each life stage using CD-HIT-EST [64,65]. Redundant contigs (percent identity > = 99%) in each cluster were eliminated, and the longest representative contigs were retained. Next, reference-based re-assembly and annotation of the contigs were performed simultaneously as described previously in two publications from this laboratory [25,50] and explained below. The parameters used in the reference-based re-assembly followed our previous publications [25,50], and were chosen to be conservative to maximize our confidence in the analyses based on the transcriptome assembly. The assembly is available at http://www.albopictusexpression.org/?q=data.

**Table 1:** Summary statistics for different stages of the hybrid assembly.

	# of contigs	N50^a^	Mean contig length^a^	Median contig length^a^	Maximum contig length^a^	Average %GC
Trinity *de novo* assembly—adult	155,321	1,866	993	497	20,723	42.03
Combined pre-adult *de novo* assemblies	539,506	1,656	972	569	23,934	42.03
Re-assembly, merged by cap3, protein reference annotated only	95,863	2,887	1,865	1,331	22,133	47.82
Re-assembly, merged by cap3, genomic reference annotated only	94,083	1,493	924	533	23,934	42.88
Complete annotated full assembly, merged by cap3	189,946	2,447	1,399.	824	23,934	46.20

^a^depicted in base pairs

### Protein reference-based re-assembly

A non-redundant dipteran protein reference set was generated by downloading orthologous protein sequences from *Ae*. *aegypti*, *Culex quinquefasciatus*, *Anopheles gambiae* and *Drosophila melanogaster* from OrthoDB [66], Version 7 (accessed on September 13th, 2013), with one single ortholog retained per ortholog group in the order specified above (i.e., order of relatedness to *Ae*. *albopictus*). The final reference set contained 19,272 protein sequences, and represented a wide range of evolutionary diversity within Diptera with little redundancy. The merged contig set from adult and pre-adult stages was first aligned to the dipteran protein set by BLASTX [67]. Alignments with e-value ≤ 1e^-6^ were retained for subsequent analysis. Contigs that aligned to the same reference and with more than 95% identity at the overlapping regions were reassembled by CAP3 [68]. To verify the annotation of the re-assembled contigs, they were again aligned to the dipteran protein set by BLASTX and only the contigs that matched the original annotation (e-value ≤ 1e^-6^) were retained. Only contigs with more than 70% identity to the matching reference were retained in the final assembly. Chimeric contigs were identified as having secondary alignments longer than 50 bp outside of the primary alignment with > 80% percent identity of the primary alignment. Chimeric contigs were discarded.

### Genome reference-based re-assembly

Contigs not retained in the protein reference-based re-assembly were subsequently used in a genome reference-based re-assembly using genomic scaffolds from *Ae*. *aegypti* [51] as a reference (accessed from VectorBase on September 30th, 2013 with SCAFFOLDS_AaegL1 for alignment and TRANSCRIPTS_AaegL1.4 for annotation). To reduce the computational requirements for alignment against the *Ae*. *aegypti* genome, contigs were first matched to the *Ae*. *aegypti* genomic scaffolds using BLASTN [67] (e-value ≤ 1e^-6^) to find the best matching scaffolds for each contig. Next, the contigs were aligned to their best matching scaffolds using EXONERATE [69] (parameters were—model est2genome—softmasktarget TRUE—bestn 1—dnahspdropoff 0). To be conservative, only the top 95% of contigs with the best alignments (percent identity > 71.78%) were retained in subsequent analysis. Contigs that aligned to the same reference with > 95% identity of the overlapping regions were re-assembled by CAP3 [68]. To verify the annotation, re-assembled contigs were re-aligned to the *Ae*. *aegypti* genomic scaffolds by EXONERATE with the same parameters as the first alignment and only the contigs that matched the original annotation were retained. Similar to the first EXONERATE alignment, the top 95% of contigs with the best alignments (percent identity > 66.67%) were retained in the final assembly. The lower percent identity of the top 95% of re-assembled and re-aligned contigs reflects the longer contig length of this set. Similar to the protein reference-based re-assembly, chimeric contigs were identified as contigs having secondary alignments outside of the primary alignment and were discarded. Contigs that aligned within 1 kb up- or down-stream of annotated gene models with at least 90% of their length covered by the alignment were identified as potential untranslated regions (UTRs) for those genes. Contigs that did not align within 1 kb up- or down-stream of annotated gene models were labeled as unannotated genomic contigs.

As a result of the procedures described above, the final transcriptome assembly contained contigs annotated based on the dipteran protein reference set, gene models in the *Ae*. *aegypti* genome, potential UTRs and unannotated *Ae*. *aegypti* genomic regions.

### Read mapping and differential gene expression analysis

The final transcriptome assembly described above was used as a reference for read mapping to quantify levels of gene expression. Cleaned paired-end reads from all 16 libraries were mapped to the annotated full assembly using RSEM ([70], Version 1.2.4) to calculate read counts at the unigene level, accounting for redundancy because of allelic variation and/or alternative splicing. Reads counts were then processed in edgeR [71] in the R software environment (www.r-project.org). First, the read counts were TMM normalized in edgeR to account for differences in library sizes and the total numbers of mRNAs sequenced across samples [71]. Genes with log counts per million smaller than one in at least four libraries were discarded as too rare for the differential expression analysis (see [72]). Differentially expressed (DE) genes were identified as having an absolute value of log_2_ fold-change greater than 0.5 with a Benjamini-Hochberg (FDR)-corrected *p*-value less than 0.05 [25,26]. Previous studies using the same population, sequencing facility, transcriptome assembly, read mapping and normalization methods indicate high correspondence (Pearson’s *r* = 0.92, 20 genes) between gene expression levels measured by RNA-Seq and qRT-PCR [25]. Distance matrices of gene expression across the libraries (R function *dist*) were visualized using multi-dimensional scaling (R function *cmdscale*) after raw read counts were transformed for linear modeling via the function *voom* [73,74] in *limma* [75,76].

### KEGG pathway enrichment analysis and additional analysis

To identify differential expression of functionally related groups of genes we tested for KEGG pathways [77,78] that were enriched for DE genes using GOSeq [79], which corrects for the selection bias of DE genes caused by transcript length. KEGG pathway assignments were downloaded from http://www.genome.jp/kegg/. We used KEGG pathway assignments from *Ae*. *aegypti*, which is in the same subgenus (*Stegomyia*) as *Ae*. *albopictus* and the most closely related species with well-documented KEGG pathway information. KEGG pathways were considered significantly enriched (i.e., over-represented), if there were five or more DE genes in the group with a FDR corrected *p*-value of over-representation less than 0.05 [25,26].

In addition to enriched KEGG pathways, we also analyzed additional pathways based on preliminary data analysis and/or *a priori* expectations concerning the molecular physiology of diapause induction. These pathways included fatty acid metabolism, DNA replication and cell cycle regulation. For DNA replication and cell cycle regulation, additional genes not included in the KEGG pathways but documented on the Interactive Fly (http://www.sdbonline.org/sites/fly/aimain/1aahome.htm) database were added to the analysis. Expression levels of all DE genes in enriched pathways were standardized as Z-scores and visualized as heat maps generated using hierarchical clustering (R function *hclust*).

## Results

### Diapause incidence

Diapause incidence ranged from 81.4% to 94.8% among three biological replicates of the females reared under diapause-inducing (SD) conditions and from 1.5% to 3.3% among three biological replicates of females reared under non-diapause-inducing (LD) conditions (S1 Table).

### Transcriptome *de novo* assembly and reference-based re-assembly

472,006,080 read pairs were obtained from the Illumina HiSeq 2000 platform of which 182,036,362 read pairs and 108,891,552 single-end reads (472,964,276 total reads) were retained after read cleaning. After digital normalization, 285,061,839 reads were utilized for *de novo* assembly via Trinity. *De novo* assembly of reads from the adult stage via Trinity produced 155,321 contigs with a median contig length of 497 bp (mean = 993 bp; Table 1). These contigs were combined with 539,506 contigs from the pre-adult stages of *Ae*. *albopictus* with a median contig length of 569 bp (mean = 972 bp). The resulting protein reference-based re-assembly with contigs merged using CAP3 produced 95,863 contigs with a median length of 1,331 bp (mean = 1,865 bp; Table 1). The median contig length for the genome reference-based re-assembly was 533 bp (mean = 924 bp). The annotated full assembly including both protein reference-based and genome reference-based contigs had a median contig length of 824 bp (mean = 1,399 bp; Table 1). Percent identity and coverage for the assemblies at various stages are presented in S1 Fig. Most contigs in the final transcriptome assembly were highly similar to the orthologous sequences in the dipteran protein reference set (S1A Fig, median percent identity = 92.86%) and the *Ae*. *aegypti* genome (S1A Fig, median percent identity = 80.25%). Contig coverage from the protein reference-based re-assembly was intermediate (S1B Fig, median coverage = 50.74%), due to the inclusion of UTRs in the contigs but not in the reference. Contig coverage from the genome reference-based re-assembly was high (S1B Fig, median coverage = 71.54%), indicating that most contigs were nearly fully utilized in the alignments to the genomic scaffolds of *Ae*. *aegypti*. Most gene models from the protein reference-based re-assembly represented in our transcriptome were nearly full-length transcripts (S1C Fig, median coverage = 73.24%). Genome reference-based re-assembly had low reference coverage (S1C Fig) because the genomic references are genomic scaffolds that are usually several hundred kilo-bases long.

14,077 non-redundant gene models were represented in the annotated full assembly, with 11,394 gene models in the protein reference-based re-assembly and another 8,636 gene models in the genome reference-based re-assembly. Of the 11,394 annotations based on the non-redundant Dipteran protein reference set, 10,296 were from *Ae*. *aegypti* proteins. 5,953 gene models were redundant between the two re-assemblies. The total of 14,077 annotated transcripts accounted for 80.9% of the annotated gene models in *Ae*. *aegypti* ([51]; 17,391 protein coding genes from the Liverpool strain AaegL1.4 from VectorBase.org).

### Read mapping and differential expression analysis

**Fig 2:**
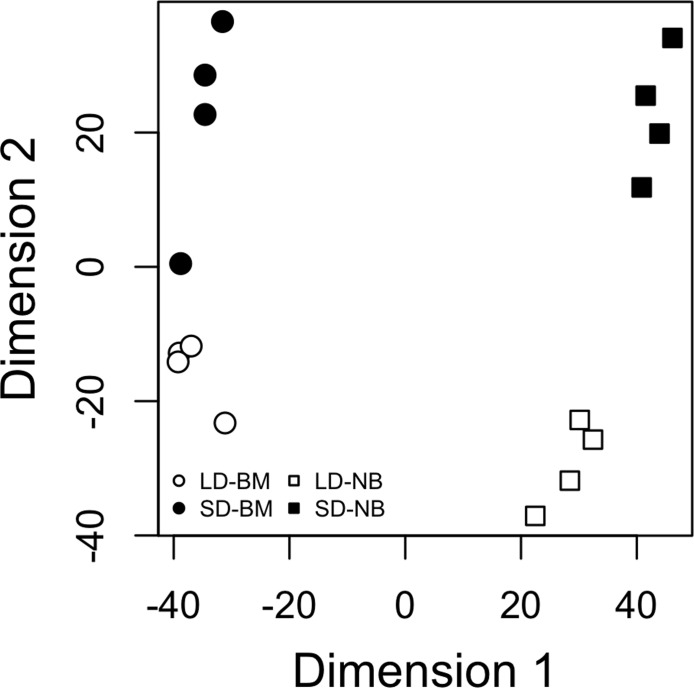
Multi-dimensional scaling plot of normalized gene expression values for photoperiodic (SD, LD) and blood feeding (BM, NB) treatments (see text for details). Two-letter symbols as in Fig 1.

Of 472,964,276 quality-filtered reads obtained from 16 libraries, we excluded unpaired single reads resulting in 182,036,362 retained read pairs (~364 million total reads). 89.44% of these read pairs mapped to the annotated full transcriptome assembly. Overall, differential gene expression profiles from biologically replicated libraries clustered tightly within each experimental treatment (Fig 2). One biological replicate from the SD blood-fed treatment exhibited increased variation relative to the other samples but was included in all downstream analyses to be conservative.

### Global transcriptional responses to a blood meal and to diapause vs. non-diapause photoperiods

**Fig 3:**
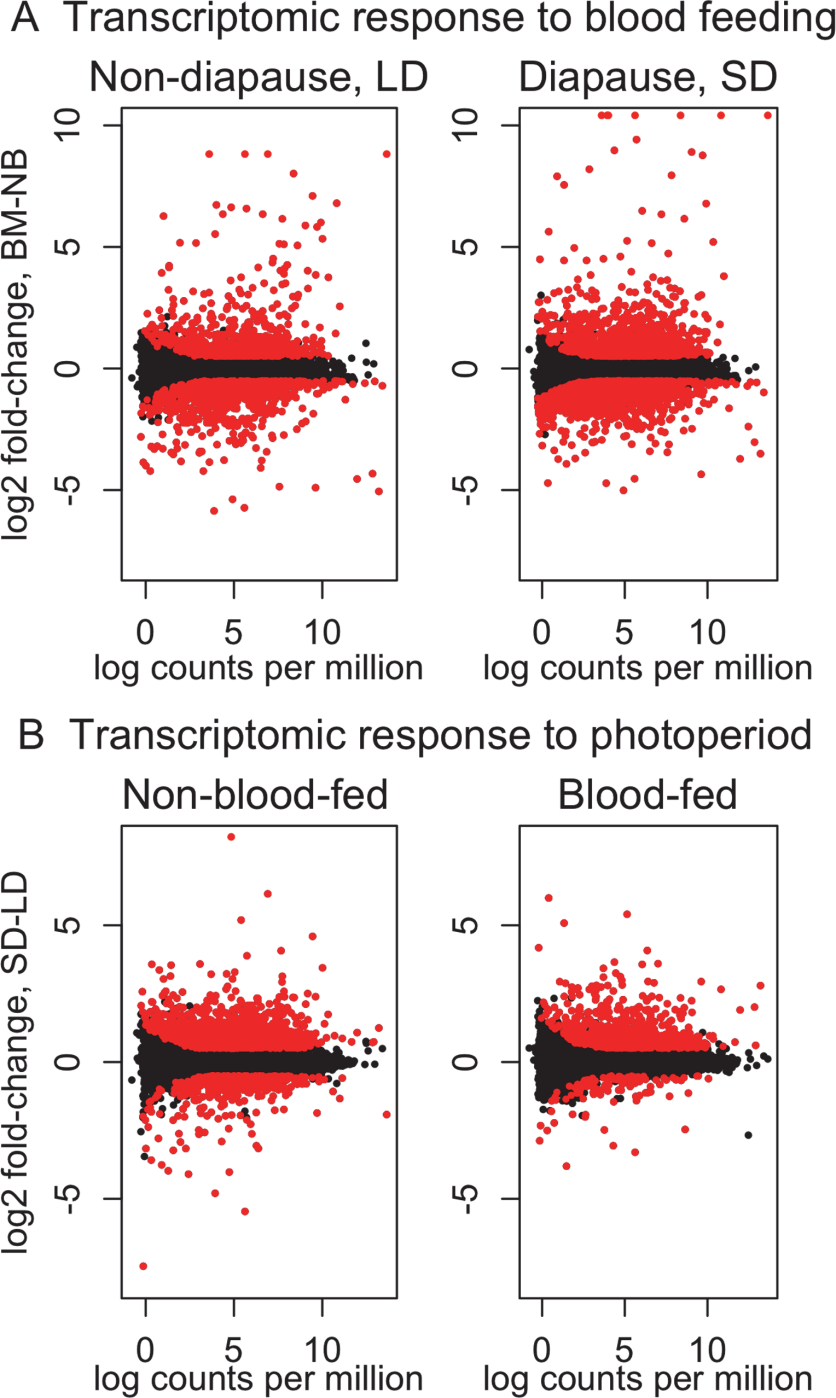
Log_2_ fold-change expression against log abundance of TMM-normalized gene expression in A) females exposed to non-diapause-inducing long day lengths (left) and diapause-inducing short day lengths (right) under blood-fed (BM) vs. non-blood-fed (NB) conditions, and B) non-blood-fed (left) and blood-fed (right) females exposed to diapause-inducing short day lengths (SD) vs. non-diapause-inducing long day lengths (LD). Each point represents an individual transcript, and positive values indicate up-regulation under blood-fed conditions (A) or diapause conditions (B). Significant differential expression (Benjamini-Hochberg corrected *P* < 0.05; absolute log_2_ fold-change > 0.5) indicated in red.

Under non-diapause-inducing LD conditions, 920 genes were significantly up-regulated in response to a blood meal (LD blood fed vs. LD non-blood fed), and 849 genes were significantly down-regulated (Fig 3A). Under diapause-inducing SD conditions, 1,566 genes were significantly up-regulated in response to a blood meal (SD blood fed vs. SD non-blood fed), and 1,408 genes were significantly down-regulated (Fig 3A). There are 665 genes that were significantly up-regulated in response to a blood meal both under SD and LD conditions, and 603 genes were significantly down-regulated in response to a blood meal both under SD and LD conditions (S2 Table).

In non-blood-fed females, 1,293 genes were significantly up-regulated under diapause-inducing SD conditions (SD non-blood fed vs. LD non-blood fed), and 524 genes were significantly down-regulated (Fig 3B). In blood-fed females, 766 genes were significantly up-regulated under diapause-inducing SD conditions (SD blood fed vs. LD blood fed), and 111 genes were significantly down-regulated (Fig 3B). There are 406 genes that were significantly up-regulated under SD vs. LD conditions in both non-blood-fed and blood-fed females, and 37 genes were significantly down-regulated under SD vs. LD conditions in both non-blood-fed and blood-fed females (S2 Table). Potential uniquely expressed genes under SD or LD with zero read count in the other photoperiodic treatment were rare in our transcriptome. This category included 184 genes of which > 97% had no more than 10 reads in any individual library. As a result, these genes were not included in the differential expression analysis. Information for all unigenes used in the differential expression analysis is presented in S2 Table.

### Differential expression in response to a blood meal

*Vitellogenin-A1 precursor* (*PVG1*) and two trypsin genes were significantly up-regulated in response to a blood meal under both diapause-inducing and non-diapause-inducing conditions (Table 2), reflecting transcriptional up-regulation of vitellogenesis and blood digestion [55,80]. *PVG1* up-regulation in response to a blood meal was greater under diapause than non-diapause conditions (blood feeding×photoperiod interaction, *p* = 0.03). Genes involved in detoxification, such as *glutathione S-transferases* (i.e., *glutathione transferases*) and *thioredoxin peroxidases* were also up-regulated in response to a blood meal (S2 Table), consistent with previous studies [81–83]. In addition, many stress response genes were differentially expressed in response to a blood meal (S2 Table).

**Table 2:** Differential expression profiles of selected genes in response to a blood meal.

Ensembl ID	Gene description	Fold change SD-BMvsSD-NB	Corrected *p*-value	Fold change LD-BMvsLD-NB	Corrected *p*-value	Corrected *p*-value interaction
AAEL010434	vitellogenin precursor A1	2483.95	1.20E-58	606.94	3.73E-46	0.03
AAEL007432	Aa SP I	1346.94	1.26E-41	259.41	1.59E-29	0.06
AAEL013284	Aa LT	71.48	6.14E-55	33.41	1.52E-40	0.14
AAEL009762	CYP307A1 (homolog of spook)	3.91	3.67E-17	2.27	2.81E-07	0.12
AAEL010946	CYP314A1 (ecdysone 20-monooxygenase)	2.12	6.16E-05	1.30	0.25	0.27
AAEL015655	CYP302A1 (ecdysteroid 22-hydroxylase)	3.14	1.38E-08	3.75	8.16E-11	0.83

Fold change and *p*-values of selected genes in response to a blood meal under diapause- and non-diapause conditions. The *p*-values for the blood feeding×photoperiod interaction are also presented, along with the Ensembl IDs and descriptions for the selected genes.

Many cytochrome P450s were also differentially expressed in response to a blood meal (S2 Table). Of special interest are *CYP302A1* and the homolog of *Spook*, both of which were up-regulated in response to a blood meal under both SD and LD photoperiods (Table 2). *CYP302A1* encodes the ecdysteroid 22-hydroxylase, a protein that catalyzes one of the final reactions in the synthesis of 20-hydroxyecdysone (20-E) and *Spook* is one of the Halloween genes implicated in synthesizing 20-E. The up-regulation of these genes in response to a blood meal is consistent with the well-established role of 20-E in stimulating vitellogenesis in response to a blood meal [55]. Finally, *CYP314A1*, which encodes ecdysone 20-monooxygenase, an enzyme catalyzing the final step in conversion of ecdysone to 20-E, was up-regulated only under diapause-inducing conditions in females with a blood meal (Table 2). In addition, all except one of the genes encoding JH-inducible proteins were down-regulated in blood-fed females (S2 Table), consistent with decreasing juvenile hormone (JH) titers after a blood meal [84].

### DE of non-blood-fed females during diapause induction

**Fig 4:**
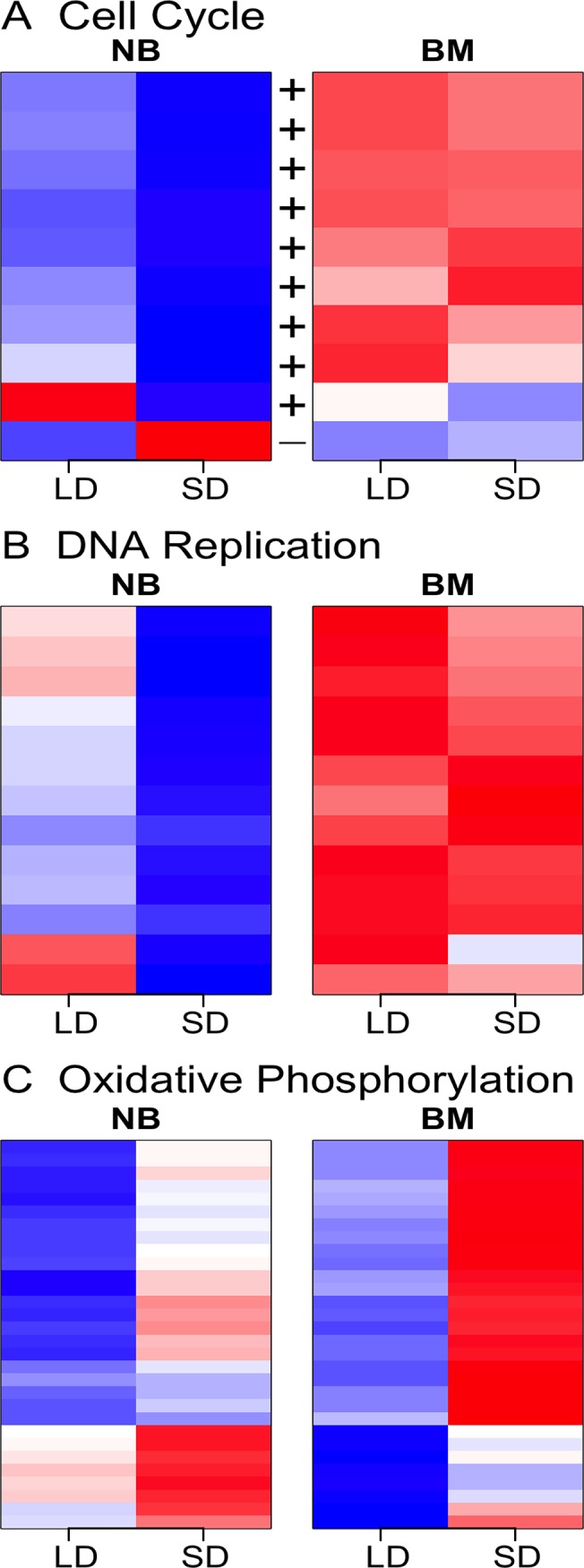
Heat maps of DE genes in the A) cell cycle, B) DNA replication and C) oxidative phosphorylation pathways under non-diapause-inducing long-day photoperiods (LD) and diapause-inducing short-day (SD) photoperiods for non-blood-fed (NB) and blood-fed (BM) females. Expression values are depicted as standardized Z-scores for each gene, where blue represents low expression and red represents high expression. “+” and “-” in Panel A indicate positive and negative cell cycle regulators, respectively.

Four KEGG pathways, three of which are related to amino acid metabolism, were enriched for differentially expressed genes under SD vs. LD conditions in females without a blood meal (Table 3). The differential expression patterns for enriched amino acid metabolism pathways under SD vs. LD conditions are summarized in a heat map (S2 Fig). The gene encoding one minor enzyme synthesizing glycine, *threonine dehydrogenase* (AAEL003443, S2 Table), was down-regulated under diapause-inducing conditions in non-blood-fed females, but genes encoding major enzymes synthesizing glycine in mammals [85] were all up-regulated under diapause-inducing conditions in non-blood-fed but not in blood-fed females (S2 Table), including *serine hydroxymethyltransferase* (AAEL002510), *sarcosine dehydrogenase* (AAEL014936) and *alanine*:*glyoxylate aminotransferase* (AAEL000640 and AAEL012464). In addition to amino acid metabolism pathways, the KEGG pathway for global metabolism was enriched for DE genes in non-blood-fed females under SD vs. LD conditions (Table 3). Similarly, all DE genes that are positive cell-cycle regulators were under-expressed under SD conditions in non-blood-fed females, and one negative cell cycle regulator, the growth arrest and DNA damage, or *GADD45*, was over-expressed (Fig 4A). Although not detected by KEGG pathway enrichment analysis, all DE genes involved in DNA replication were down-regulated under SD conditions in non-blood-fed females (Fig 4B). Consistent down-regulation in these two KEGG pathways related to cell proliferation indicates that under diapause-inducing SD conditions non-blood-fed females down-regulate cell proliferation.

**Table 3:** Representative enriched KEGG pathways under diapause conditions.

Treatment	Functional Category	KEGG_ID	Enrichment *p*-value
SD-NBvsLD-NB	Alaline, aspartate and glutamate metabolism	aag00250	5.00E-04
SD-NBvsLD-NB	Glycine, serine and threonine metabolism	aag00260	5.00E-04
SD-NBvsLD-NB	Biosynthesis of amino acids	aag01230	5.00E-04
SD-NBvsLD-NB	Metabolic pathway	aag01100	1.00E-03
SD-BMvsLD-BM	Oxidative phosphorylation	aag00190	5.00E-04
SD-BMvsLD-BM	Valine, leucine and isoleucine degradation	aag00280	1.50E-03
SD-BMvsLD-BM	beta-Alanine metabolism	aag00410	5.00E-04
SD-BMvsLD-BM	Metabolic pathway	aag01100	5.00E-04

Two-letter symbols (SD, LD, BM, NB) are as described in Fig 2. KEGG pathway IDs are specific to *Ae*. *aegypti*.

The clock genes *timeless* and *cryptochrome 1* were up-regulated under SD conditions in non-blood-fed females (Table 4) but *period* (AAEL008141) and *clock* (AAEL012562) were not differentially expressed (S2 Table). Similarly, *phosphoenolpyruvate carboxykinase* (*pepck*) was also up-regulated under SD conditions only in non-blood-fed females (significant blood feeding×photoperiod interaction, Table 4). *Delta(9)-desaturase* and *delta(9)-desaturase 2* were up-regulated under SD conditions in non-blood-fed females (Table 4). Out of eight differentially expressed genes encoding putative JH-inducible proteins under SD conditions, seven genes were up-regulated in non-blood-fed females (Table 4). The oxidative phosphorylation pathway was not significantly enriched under SD conditions in non-blood-fed females (but see below), but all 7 DE genes were up-regulated (Fig 4C).

**Table 4:** Differential expression profiles of selected genes in response to photoperiod.

Ensembl ID	Gene description	Fold change SD-NBvsLD-NB	Corrected *p*-value	Fold change SD-BMvsLD-BM	Corrected *p*-value	Corrected *p*-value interaction
AAEL006411	timeless	1.69	2.62E-06	1.30	0.06	0.32
AAEL004146	cryptochrome 1	1.48	0.04	1.05	0.88	0.44
AAEL000006	phosphoenolpyruvate carboxykinase	4.22	5.92E-05	1.38	0.52	0.10
AAEL000080	phosphoenolpyruvate carboxykinase	5.67	6.81E-13	1.09	0.83	8.13E-06
AAEL007213	delta(9)-desaturase	1.52	0.04	1.28	0.33	0.80
AAEL004573	delta(9)-desaturase 2	2.78	1.39E-04	1.96	0.04	0.69
AAEL001194	fatty acid synthase	0.86	0.35	2.31	8.12E-11	4.48E-07
AAEL007516	fatty acid desaturase	0.77	0.42	2.12	0.02	0.04
AAEL001899	juvenile hormone-inducible protein, putative	1.88	2.67E-03	1.38	0.24	0.59
AAEL003954	juvenile hormone-inducible protein, putative	1.93	2.42E-04	2.06	2.83E-04	0.93
AAEL004023	juvenile hormone-inducible protein, putative	1.29	0.09	1.50	0.01	0.73
AAEL004242	juvenile hormone-inducible protein, putative	2.64	5.82E-06	1.33	0.35	0.12
AAEL006605	juvenile hormone-inducible protein, putative	1.54	0.03	1.28	0.35	0.79
AAEL012680	juvenile hormone-inducible protein, putative	2.14	3.95E-12	1.45	0.01	0.11
AAEL014439	juvenile hormone-inducible protein, putative	1.43	0.01	1.58	2.28E-03	0.85
AAEL014440	juvenile hormone-inducible protein, putative	1.42	0.01	1.08	0.71	0.35

Fold change and *p*-values of selected genes in response to photoperiod in non-blood-fed and blood-fed females. The *p*-values for the photoperiod×blood feeding interaction are also presented, along with the Ensembl IDs and descriptions for the selected genes.

### DE of blood-fed females during diapause induction

In contrast to non-blood-fed females under SD conditions, in blood-fed females under SD conditions the oxidative phosphorylation KEGG pathway was significantly enriched (Table 3), with all DE genes up-regulated (Fig 4C). The proportion of up-regulated genes (29/89) in the oxidative phosphorylation pathway in blood-fed females was significantly higher than that (7/89) in non-blood-fed females (Fisher’s exact test, *p*-value = 1.93e-05).

Similar to non-blood-fed females under SD conditions, in blood-fed females under SD conditions the KEGG pathway for global metabolism was enriched for DE genes (Table 3). The proportion of up-regulated genes in the pathway under SD conditions in non-blood-fed females (108/665) did not differ significantly from that in blood-fed females (89/665) (Fisher’s exact test, *p*-value = 0.16), but more genes involved in metabolism were down-regulated under SD conditions in non-blood-fed females (31/665) than in blood-fed females (5/665) (S3 Fig; Fisher’s exact test, *p*-value = 1.00e-05).

*Fatty acid synthase*, *fatty acid desaturase* and *delta(9)-desaturase 2* were up-regulated under diapause-inducing conditions in blood-fed females but not in non-blood-fed females (Table 4). Similar to non-blood-fed females under SD conditions, two amino acid metabolism pathways were significantly enriched under SD conditions in blood-fed females (Table 3). In the valine, leucine and isoleucine degradation pathway, *branched-chain amino acid (BCAA) aminotransferase* (AAEL007909, S2 Table) was up-regulated under diapause-inducing conditions both in non-blood-fed and blood-fed females. In the beta-alanine metabolism pathway, one enzyme involved in synthesizing beta-alanine from uracil in insects, *dihydropyrimidine dehydrogenase* [86], was up-regulated both in non-blood-fed and blood-fed females under SD conditions (AAEL014199 and AAEL010204, S2 Table). Out of eight differentially expressed genes encoding putative JH-inducible proteins under SD conditions, four genes were up-regulated in blood-fed females (Table 4).

## Discussion

*Aedes albopictus* is a vector of increasing public health concern due to its rapid global range expansion and ability to transmit DENV, CHIKV, and at least 22 additional arboviruses [10,14]. Traditional vector control strategies such as source reduction and insecticides have been largely ineffective against *Ae*. *albopictus* [15], but targeting the molecular regulation of photoperiodic diapause or diapause-associated physiological processes could provide the basis of novel approaches to population suppression. Photoperiodic diapause is a crucial ecological adaptation in a wide range of temperate insects [17,87], but our knowledge of the molecular underpinnings of this trait is extremely limited, especially at the diapause induction phase. In this study, we examined genome-wide transcriptional dynamics during diapause induction in blood-fed and non-blood-fed *Ae*. *albopictus* adult females. This study is the first to utilize powerful RNA-Seq technologies to elucidate the molecular mechanisms underlying diapause induction at the transcriptome level in any insect (but see an integrated proteomic and metabolomic analysis of diapause induction by [88]). The highly divergent diapause response of eggs from females exposed to SD vs. LD (S1 Table) and the generally consistent gene expression profiles of biological replicates within each experimental treatment (Fig 2) indicate that the F_3_ generation used in this experiment a had robust and consistent diapause response at both the phenotypic and transcriptional level.

### Transcriptome assembly and annotation

We combined 155,321 contigs obtained in this study from the adult life stage with 539,506 contigs obtained previously from pre-adult stages to produce a composite transcriptome assembly (Table 1). The number of gene models identified from the current comprehensive assembly including the adult stage increased to 14,077 from 13,261 gene models identified based on the pre-adult stages [50]. These 14,077 gene models represent approx. 81% of all annotated gene models in *Ae*. *aegypti* (AaegL1.4). The annotated comprehensive assembly is also moderately improved relative to previous assemblies [25,50] in terms of median contig length, contig coverage and gene model coverage (Table 1 and S1 Fig). Based on comparison with the *Ae*. *aegypti* genome, this comprehensive transcriptome assembly likely represents the majority of the coding regions in the *Ae*. *albopictus* genome and therefore provides a powerful resource to effectively investigate global transcriptional components of diapause induction.

### Transcriptional responses to a blood meal

Similar to our results, a previous RNA-Seq analysis of the transcriptional response to a blood meal in the closely related mosquito *Ae*. *aegypti* found extensive differential gene expression [89]. However, because adult females in this previous study [89] were maintained at 28°C rather than 21°C and RNA was extracted at 5 hours rather than 26–28 hours post blood meal, our results are not directly comparable. Nevertheless, extensive additional information on the transcriptional response to a blood meal in *Ae*. *aegypti* under LD conditions allows us to validate the transcriptional response to blood feeding in *Ae*. *albopictus*. For example, vitellogenin synthesis and blood digestion are key physiological components of the transcriptional response to a blood meal [55]. Both vitellogenin synthesis and typsin activity reach their peak at approx. 24 hours post blood meal in *Ae*. *aegypti* maintained at 27°C [80,90]. Populations used in this study were maintained at 21°C in order to optimally stimulate a robust diapause response, and thus the peaks of vitellogenin synthesis and blood digestion likely would have occured after 24 hours pbm. Therefore, the time point of sampling in this study, 26–28 hours pbm, is expected to correspond to near the peak of vitellogenin synthesis and blood digestion, consistent with the highly elevated transcriptional profiles of *PVG1* and *trypsins* in response to a blood meal (Table 2). 20-hydroxyecdysone (20-E) is an essential hormone stimulating vitellogenesis after females take a blood meal [55]. We found that three genes encoding enzymes in the 20-E synthesis pathway [91] were up-regulated in response to a blood meal (Table 2). Juvenile hormone also plays a crucial role in regulating the reproduction of adult mosquitoes [55]. In *Ae*. *aegypti*, after females take a blood meal, JH titers decrease until the end of a gonotrophic cycle [84], in antiphase with 20-E [55]. Consistent with this pattern, our results show that 13 out of the 14 DE genes encoding putative JH-inducible proteins were down-regulated in response to a blood meal under either diapause-inducing or non-diapause-inducing conditions (S2 Table).

Cytochrome P450 (CYP) monooxygenases are mainly involved in hormone synthesis and insecticide resistance [92]. All three 20-E synthesizing CYP enzymes noted above (Table 2) were up-regulated in response to a blood meal, consistent with the role of 20-E in promoting vitellogenesis. Additionally, *glutathione S-transferases* and *thioredoxin peroxidases* are mostly up-regulated in response to a blood meal (S2 Table), potentially as a response to oxidative stress since these enzymes remove intracellular reactive oxygen species. It is also possible that the glutathione transferases might be involved in heme detoxification [93]. Finally, six out of seven genes involved in response to water stress were down-regulated in response to a blood meal. These results are generally consistent with a previous study in the *Ae*. *aegypti* midgut [83] and in the *Ae*. *albopictus* malpighian tubules [94]. These genes are likely related to osmotic stress and the intake of toxic substances (i.e., heme) or microbes associated with blood feeding.

Overall, the transcriptional responses to a blood meal detected in this study are consistent with previous studies and support the conclusion that transcriptome sequencing of whole bodies captured the major physiological benchmarks of the response to a blood meal. Furthermore, the overall level of differential expression in response to a blood meal was similar under SD and LD conditions. Under SD conditions, 11.2% more genes were up-regulated than down-regulated in response to a blood meal. Under LD conditions, 8.4% more genes were up-regulated than down-regulated.

### Transcriptional changes during diapause induction in non-blood-fed females

We hypothesized that some of the “upstream” transcriptional components of diapause induction would occur before adult female *Ae*. *albopictus* obtain access to a blood meal. Consistent with hypothesis, we found that *timeless* (*tim*) and *cryptochrome 1* (*cry1*), two essential components of the circadian clock pathway in insects [39], were up-regulated under diapause-inducing conditions in non-blood-fed females (Table 4). Because we controlled for circadian effects on gene expression by harvesting female whole bodies at the same Zeitgeber time, the increased expression of *tim* and *cry1* under SD relative to LD conditions is interpreted as a response to diapause-inducing photoperiods. For almost 80 years, researchers have hypothesized that the circadian clock constitutes the underlying molecular mechanism for photoperiodic time measurement [95]. However, the causative link between these two biological timing systems remains unresolved and controversial [38,42]. In both *Anopheles gambiae* and *Ae*. *aegypti*, under LD, *tim* decreases at ZT 6–8h [96,97] relative to earlier and later peaks in the 24-hour cycle. In contrast, *cry1* increases at ZT 6–8h in *An*. *gambiae* [97], consistent with the role of CRY1 in the light-dependent degradation of TIM [39]. Among dipteran species, *tim* is required for diapause induction of *Chymomyza costata* [98,99]. At ZT 6–8h, the transcript level of *tim* is up-regulated both in the photosensitive larval brain of *Sarcophaga crassipalpis* under diapause-inducing conditions [100] and in the diapausing *Wyeomyia smithii* fourth instar larvae [101]. In *Drosophila triauraria*, additive allelic differences in *tim* and *cry1* between diapause and non-diapause strains are positively associated with diapause incidence [102].

As a fundamental physiological timekeeper, the circadian clock system is responsible for the rhythmic expression patterns of thousands of genes throughout the 24-hour daily cycle [97,103]. Therefore, abnormal expression patterns in the circadian clock system are expected to cause considerable disruptions in the expression of genes important for a wide range of physiological functions other than diapause. Hence, it has been proposed that *tim* may be functionally involved in measuring photoperiodic (seasonal) time independent of its role in the circadian clock [42,104]. Our results are consistent with this hypothesis. In addition to *tim*, *period* (*per*) and *clock* (*clk*) are core components of the transcriptional negative feedback loop that drives the oscillatory behavior of the circadian clock [39]. Differential expression of *tim* but not *per* and *clk* in response to diapause-inducing short day lengths suggests that short-day photoperiods do not cause fundamental changes to the oscillatory behavior of the circadian clock. Because CRY1 is responsible for the light-sensitive degradation of TIM, we hypothesize that products of the CRY1-mediated breakdown of TIM could serve as a component of an “interval” photoperiodic timer in *Ae*. *albopictus*, independent of the circadian clock pathway.

Two amino acid metabolism KEGG pathways were significantly enriched for differentially expressed genes in non-blood-fed females under diapause-inducing conditions: A) alanine, aspartate and glutamate metabolism, and B) glycine, serine and threonine metabolism (Table 3). Alanine levels increase during diapause initiation in *Teleogryllus emma* [105], *B*. *mori* [106,107], and *Ostrinia furnacalis* [108]. Alanine levels also increase during diapause in *S*. *crassipalpis* [109], and both before and during diapause in *Antheraea pernyi* [110]. In *A*. *pernyi*, alanine levels also decrease as diapause terminates [110]. Despite this widespread association of alanine with the diapause program in a broad range of insects, the biological significance has not been elucidated. In this study, *alanine aminotransferase* (AAEL009872) and *alanine-glyoxylate aminotransferase* (AAEL000640) were up-regulated under diapause-inducing conditions in NB females (S2 Table), suggesting increased metabolism of alanine. We hypothesize that alanine may be provisioned to the diapause offspring from the mother and could serve as a cryoprotectant as has been proposed for the diapause eggs of *B*. *mori* [107].

Inspection of genes in the glycine, serine and threonine metabolism KEGG pathway indicates that the gene encoding a minor enzyme synthesizing glycine, *threonine dehydrogenase*, was down-regulated under diapause conditions in non-blood-fed females. However, genes encoding major enzymes synthesizing glycine in mammals [85] were all up-regulated, including *serine hydroxymethyltransferase* (AAEL002510), *sarcosine dehydrogenase* (AAEL014936) and *alanine*:*glyoxylate aminotransferase* (AAEL000640) (S2 Table). Diapause-destined larvae of *Helicoverpa armigera* accumulate more glycine [111]. In *Leptinotarsa decemlineata*, glycine-rich transcripts are up-regulated during diapause initiation phase [112]. Glycine has been implicated to regulate protein synthesis in vertebrates, and it could also regulate growth and development by serving as an indicator of nutrient levels [85].

In non-blood-fed *Ae*. *albopictus* adults during diapause induction, both positive cell cycle regulators and DNA replication transcripts were down-regulated (Fig 4A and 4B), particularly the positive cell cycle regulator *proliferating cell nuclear antigen* (*pcna*). Transcriptional suppression of the cell cycle is a common molecular hallmark of the diapause program during the developmental arrest stage of diapause, as illustrated in *S*. *crassipalpis* [113,114], *Helicoverpa armigera* [115] and *C*. *costata* [116]. Furthermore, *pcna* is down-regulated during diapause both in *S*. *crassipalpis* [113,114], and *C*. *costata* [116]. However, cell cycle transcripts are up-regulated after diapause termination in *Rhagoletis pomonella* [28]. The *pcna* transcript is up-regulated after diapause termination both in *S*. *crassipalpis* [113] and *R*. *pomonella* [28], in synchrony with other changes in cell cycle regulation. In the current study we examined the adult stage which represents diapause induction rather than developmental arrest. Thus, our results are likely not relevant to cessation of the development during diapause. Rather, we hypothesize that cell proliferation is down-regulated under diapause-inducing conditions before females take a blood meal to allocate energy to alternative metabolic pathways. This interpretation is consistent with results from the oxidative phosphorylation pathway discussed below. These results are also consistent with a previous study showing alteration of the cell cycle during diapause preparation in early *Ae*. *albopictus* embryos [25] and emphasize that diapause induction involves the alteration of fundamental cellular processes far in advance of developmental arrest.

The *pepck* transcript was up-regulated under diapause-inducing condition in non-blood-fed but not blood-fed females (significant interaction, Table 4). The up-regulation of *pepck* is similar to previous studies in mature oocytes, developing embryos and pharate larvae under diapause conditions in *Ae*. *albopictus* [24–26]. The *pepck* transcript is also up-regulated under diapause conditions in several other insect species, including *S*. *crassipalpis* [27], and *R*. *pomonella* [28], as well as in the dauer phenotype (the counterpart of diapause in nematodes) of *Caenorhabditis elegans* [117]. In *W*. *smitthii*, up-regulation of *pepck* is associated with diapause termination [118]. The *pepck* transcript is involved in response to cold and desiccation in *Belgica antarctica* [119] and in response to hormone stimulation in *Drosophila* [120]. It is also down-regulated by nectarine supplemented diet which increases longevity in *Drosophila* [121]. The observation that *pepck* was differentially expressed under diapause-inducing conditions only in non-blood-fed females suggests *pepck* could be a regulatory component of pre-diapause metabolism, potentially triggering a cascade of metabolic responses after the females take a blood meal. In light of its association with the diapause program across multiple stages in *Ae*. *albopictus*, with diapause induction or termination phases in other insects, and with stress resistance and hormonal response, it is increasingly evident that *pepck* is a central component of diapause metabolism in a wide range of organisms.

Juvenile hormone has been implicated in the regulation of larval diapause in a variety of species [36] and the absence of JH has been demonstrated to initiate adult reproductive diapause in *Cx*. *pipiens* [33–35]. However, a role of JH in pharate larval diapause has not been noted in previous studies. For *Ae*. *albopictus*, in non-blood-fed females all seven differentially expressed genes encoding putative JH-inducible proteins were up-regulated under SD conditions (Table 4). Before a blood meal, JH induces the primary follicles to enter a resting stage, and also renders the fat body competent for vitellogenin synthesis after a blood meal [55]. Increased JH-induced signaling under diapause conditions in non-blood-fed females likely enhances the fat body’s competence for vitellogenin synthesis after a blood meal, thereby increasing vitellogenesis for augmented nutrient provisioning to offspring destined to undergo diapause. In blood-fed females under SD conditions, all four differentially expressed genes encoding putative JH-inducible proteins were up-regulated (Table 4). Under non-diapause conditions, JH levels are expected to decrease after a blood meal [84]. Therefore, the up-regulation of JH-induced signaling under diapause conditions in blood-fed females implies altered reproductive endocrinology during diapause induction.

### Transcriptional changes during diapause induction in blood-fed females

Energy metabolism is crucial for the survival of diapause insects through the winter. Levels of nutrient reserves during diapause directly affect overwinter survival, as well as post-diapause development and reproduction [29]. Photoperiodic diapause is determined maternally in *Ae*. *albopictus*, and diapause offspring (pharate larvae inside the egg) cannot obtain additional resources. As a result, maternal provisioning of diapause eggs is expected to have a large impact on offspring fitness. In fact, previous studies have established that diapause eggs of *Ae*. *albopictus* are larger and contain more total lipids than non-diapause eggs [49]. Consistent with these considerations, energy production (oxidative phosphorylation) and overall metabolism were elevated under SD conditions in blood-fed females (Fig 4C and S3 Fig). Overall, our study suggests that after taking a blood meal, females exposed to SD signals enhance energy production through the oxidative phosphorylation pathway, presumably to meet the energetic requirements for generating more nutrients to provision the offspring destined to undergo diapause as described below.

Analysis of individual genes up-regulated in blood-fed females under SD conditions provides further insight into the molecular basis of provisioning of diapause eggs. For example, up-regulation of vitellogenin synthesis gene *PVG1* in response to a blood meal is greater under SD than LD conditions (significant interaction, Table 2). Additionally, *fatty acid synthase* (*fas*) was up-regulated under SD conditions only in blood-fed females (significant interaction, Table 4), indicating that blood-fed females synthesize more fatty acids under diapause-inducing than non-diapause-inducing conditions. This is consistent with previous results stated above that diapause eggs contain more total lipids compared to non-diapause eggs [49]. In *Cx*. *pipiens*, *fas* is elevated in diapause-destined females that overwinter at the adult stage, consistent with our results and the general importance of lipids as nutrient reserves during diapause [29]. Three genes encoding fatty acid desaturases were also up-regulated under SD conditions (significant interaction in *fatty acid desaturase*, Table 4), indicating that synthesis of desaturated fatty acids (UFAs) was increased in females exposed to diapause-inducing conditions. UFAs are proposed to enhance cold tolerance during diapause by preserving membrane permeability under low temperatures [122]. Our results are consistent with the previous studies that fatty acid desaturation is enhanced under diapause conditions [29,123] and cold acclimation [124]. These results imply that maternal provisioning of UFAs to the offspring contribute to the increased cold tolerance of diapause relative to non-diapause eggs in *Ae*. *albopictus* [46].

Two amino acid metabolism pathways were significantly enriched in blood-fed females under diapause-inducing conditions: valine, leucine and isoleucine degradation, as well as beta-alanine metabolism (Table 3). Michaud and Denlinger [109] reported increased leucine during the pupal diapause of *S*. *crassipalpis*. In mammals, leucine stimulates protein synthesis [125], but no research has been performed regarding the effect of leucine on diapause in invertebrates. However, in the light of enhanced vitellogenesis in females exposed to SD with a blood meal, increased leucine might stimulate more protein synthesis after a blood meal to provision the diapause offspring. Beta-alanine has been implicated to recycle the photoreceptor neurotransmitter histamine in the photoreceptor cells of *Drosophila* [126]. One gene involved in synthesizing beta-alanine from uracil in insects, *dihydropyrimidine dehydrogenase* [86], was up-regulated both in non-blood-fed and blood-fed females under diapause conditions (S2 Table). The role of beta-alanine has not been examined in terms of diapause response, but increased beta-alanine under SD conditions might be used for differentially measuring photoperiod via its ability to recycle histamine, the photoreceptor neurotransmitter in insects.

### Conclusions and Significance

Diapause is an adaptive developmental plasticity of crucial ecological importance. Our results show that thousands of genes are differentially expressed under diapause-inducing conditions (Fig 3B), but only approximately 1% of all genes are potentially uniquely expressed under diapause-inducing or non-diapause-inducing conditions. Therefore our study indicates that the transcriptional basis of diapause induction is primarily a quantitative rather than qualitative response, with changes involving mostly levels of transcription rather than specific genes that are uniquely expressed under either diapause or non-diapause conditions. This conclusion implies that a wide range of fundamental physiological pathways modified as part of the diapause response may also provide novel targets for genetic or chemical disruption under non-diapause conditions. We have identified novel putative regulatory elements of diapause induction (i.e., *tim* and *cry1*), and our study confirms previous hallmarks of insect diapause at the transcriptional level, such as cell cycle regulation, *pepck* and lipid metabolism. Diapause appears to have evolved independently in several lineages within both Culicidae [19] and Diptera [127]. Our study supports a previous hypothesis [25] that despite hundreds of millions of years of evolution among dipteran species, a conserved set of genes has been repeatedly targeted by selection during the evolution of diapause in independent lineages, including *pepck*, *pcna* and *fas*. These genes provide targets for functional studies aimed at developing novel control strategies designed to disrupt the photoperiodic diapause response, a crucial ecological adaptation in a wide range of pest and vector species.

Below is a list of the genes mentioned in the text and their Ensembl IDs, in the order of their appearance:

*vitellogenin-A1 precursor*, AAEL010434; *serine protease I*, AAEL007432; *late trypsin*, AAEL013284; *glutathione S-transferases*/*glutathione transferases*, AAEL000092, AAEL001061, AAEL001090, AAEL004229, AAEL007947, AAEL007955, AAEL007964, AAEL010157, AAEL010582, AAEL010591, AAEL011741, AAEL011752, AAEL011934, CPIJ018630; *thioredoxin peroxidases*, AAEL002309, AAEL004112, AAEL014548; *CYP302A1*, AAEL015655; *Spook*, AAEL009762; *CYP314A1*, AAEL010946; *threonine dehydrogenase*, AAEL003443; *serine hydroxymethyltransferase*, AAEL002510; *sarcosine dehydrogenase*, AAEL014936; *alanine*:*glyoxylate aminotransferase*, AAEL000640, AAEL012464; *growth arrest and DNA damage*, or *GADD45*, AAEL006883; *timeless*, AAEL006411; *cryptochrome 1*, AAEL004146; *period*, AAEL008141; *clock*, AAEL002049; *phosphoenolpyruvate carboxykinase*, AAEL000006, AAEL000080; *delta(9)-desaturase*, AAEL007213; *delta(9)-desaturase 2*, AAEL004573; *fatty acid synthase*, AAEL001194; *fatty acid desaturase*, AAEL007516; *branched-chain amino acid aminotransferase*, AAEL007909; *dihydropyrimidine dehydrogenase*, AAEL014199, AAEL010204; *alanine aminotransferase*, AAEL009872; *proliferating cell nuclear antigen*, AAEL012545.

## Supporting Information

S1 Fig Representation of alignments in protein-based and genome-based re-assembly. Percentage identity (a), proportion of contig in the alignment (b) and proportion of reference in the alignment (c) resulting from alignments of contigs from composite transcriptome assembly to the protein and genomic references.
(PNG)

S2 Fig Amino acid metabolisms pathways under LD and SD in BM and NB females. Heat maps of DE genes in the enriched amino acid metabolism pathways (Table 3). Symbols and conventions as in Fig 4.
(PNG)

S3 Fig Global metabolic pathway under LD and SD in BM and NB females. Heat maps of DE genes in the global metabolic pathway. Symbols and conventions as in Fig 4.
(PNG)

S1 Table Diapause incidence of the Manassas, VA population used in this study. (XLSX)

S2 Table Expression and annotation information for all unigenes in the analysis. (XLSX)
