# Supplementary figures and images for: Global Transcriptional Dynamics of Diapause Induction in Non-Blood-Fed and Blood-Fed Aedes albopictus
Source: PLoS Negl Trop Dis. 2015 Apr 21;9(4):e0003724. doi: 10.1371/journal.pntd.0003724 (PMC4405372; doi:10.1371/journal.pntd.0003724)

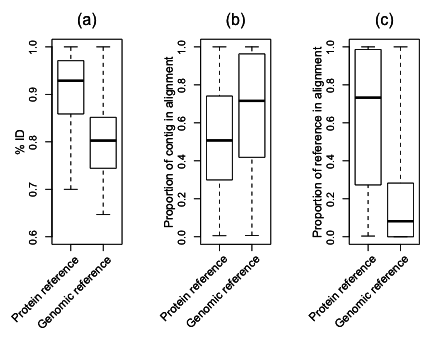

Supplement: S1 Fig — Percentage identity (a), proportion of contig in the alignment (b) and proportion of reference in the alignment (c) resulting from alignments of contigs from composite transcriptome assembly to the protein and genomic references. (PNG) [file pntd.0003724.s001.png]

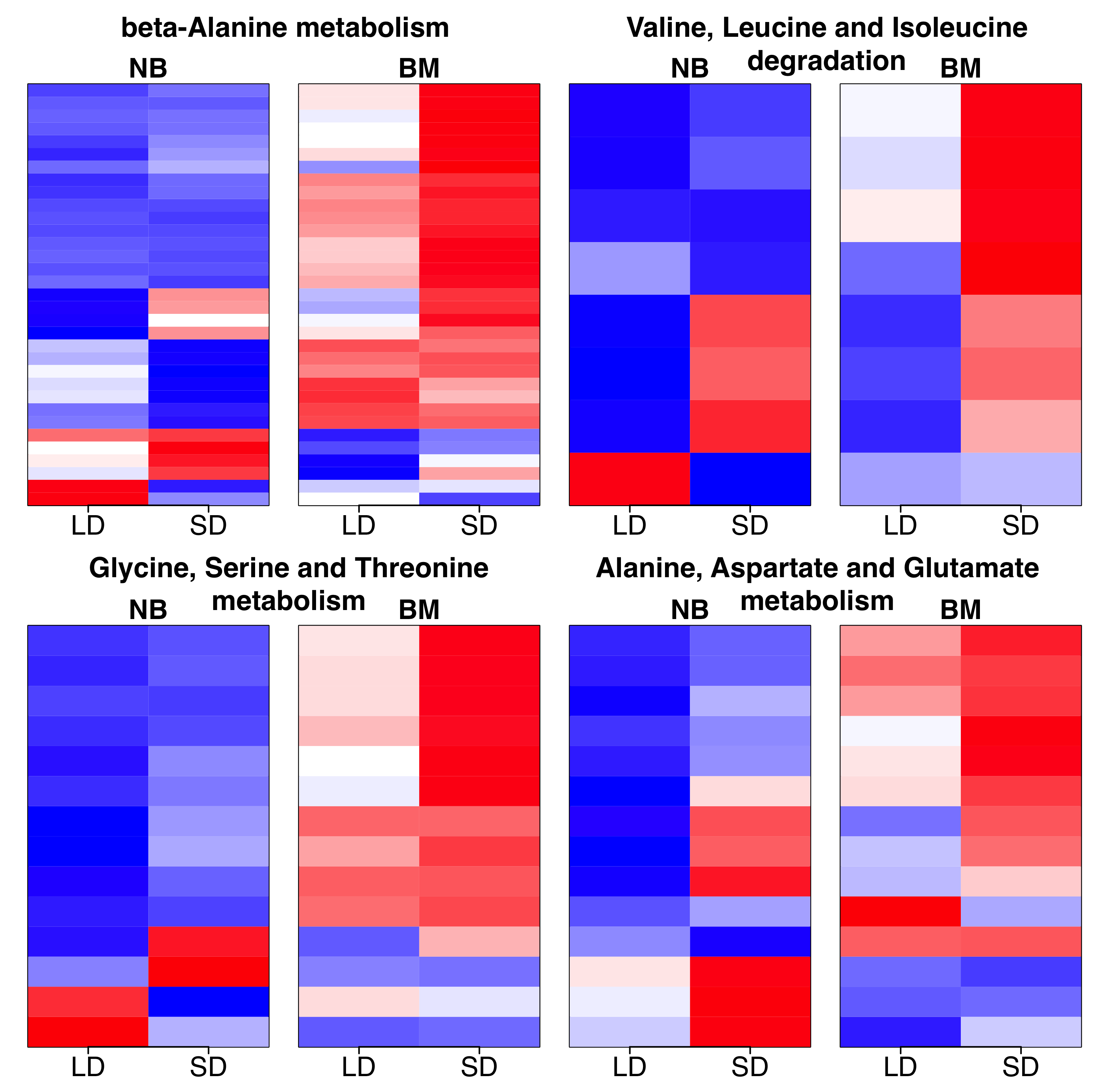

Supplement: S2 Fig — Heat maps of DE genes in the enriched amino acid metabolism pathways (Table 3). Symbols and conventions as in Fig 4. (PNG) [file pntd.0003724.s002.png]

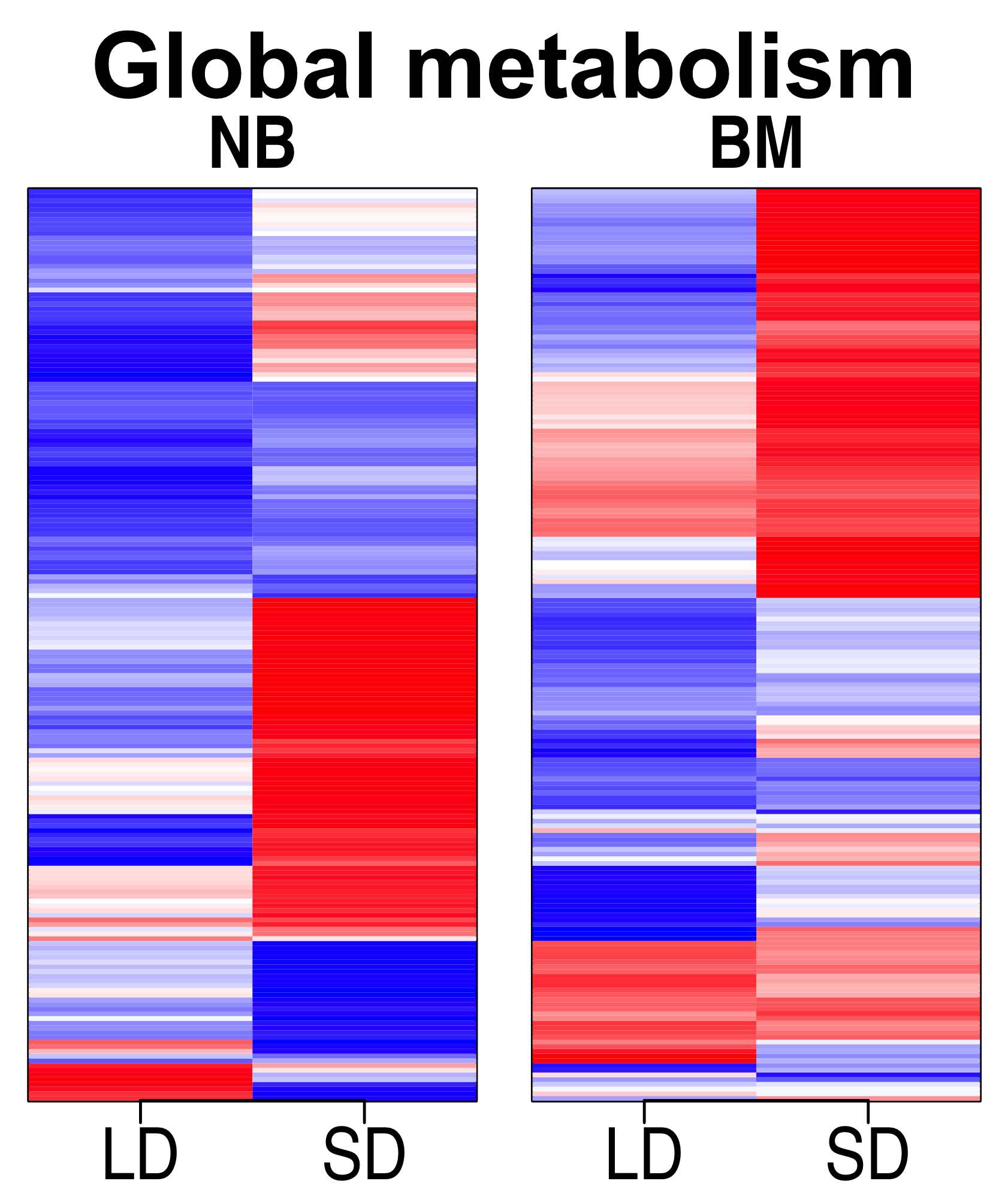

Supplement: S3 Fig — Heat maps of DE genes in the global metabolic pathway. Symbols and conventions as in Fig 4. (PNG) [file pntd.0003724.s003.png]
